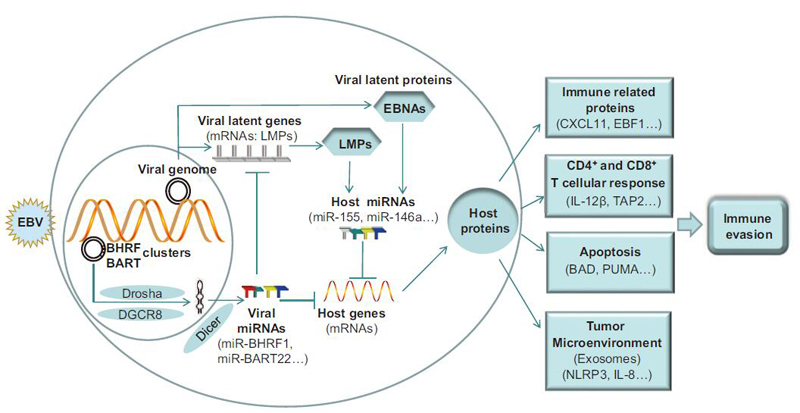# An update: Epstein-Barr virus and immune evasion *via* microRNA regulation

**DOI:** 10.1007/s12250-017-3996-5

**Published:** 2017-06-26

**Authors:** Lielian Zuo, Wenxin Yue, Shujuan Du, Shuyu Xin, Jing Zhang, Lingzhi Liu, Guiyuan Li, Jianhong Lu

**Affiliations:** 1 The Key Laboratory of Carcinogenesis of the Chinese Ministry of Health, Xiangya Hospital, Central South University, Changsha, 410080 China; 2 Cancer Research Institute, School of Basic Medical Sciences, Central South University, Changsha, 410078 China; 3 Department of Microbiology, School of Basic Medical Sciences, Central South University, Changsha, 410078 China

**Keywords:** Epstein-Barr virus (EBV), microRNAs, immune evasion, exosomes, carcinogenesis

## Abstract

Epstein-Barr virus (EBV) is an oncogenic virus that ubiquitously establishes life-long persistence in humans. To ensure its survival and maintain its B cell transformation function, EBV has developed powerful strategies to evade host immune responses. Emerging evidence has shown that microRNAs (miRNAs) are powerful regulators of the maintenance of cellular homeostasis. In this review, we summarize current progress on how EBV utilizes miRNAs for immune evasion. EBV encodes miRNAs targeting both viral and host genes involved in the immune response. The miRNAs are found in two gene clusters, and recent studies have demonstrated that lack of these clusters increases the CD4^+^ and CD8^+^ T cell response of infected cells. These reports strongly indicate that EBV miRNAs are critical for immune evasion. In addition, EBV is able to dysregulate the expression of a variety of host miRNAs, which influence multiple immune-related molecules and signaling pathways. The transport via exosomes of EBV-regulated miRNAs and viral proteins contributes to the construction and modification of the inflammatory tumor microenvironment. During EBV immune evasion, viral proteins, immune cells, chemokines, pro-inflammatory cytokines, and pro-apoptosis molecules are involved. Our increasing knowledge of the role of miRNAs in immune evasion will improve the understanding of EBV persistence and help to develop new treatments for EBV-associated cancers and other diseases.

## INTRODUCTION

Epstein-Barr virus (EBV) was the first confirmed oncogenic virus capable of infecting humans, and it belongs to the human herpesvirus family. It is associated with several lymphocytic and epithelial malignancies in humans such as Burkitt's lymphoma and nasopharyngeal carcinoma (NPC) (Young and Rickinson, 2004; Luo and Ou, 2015; Tsang and Tsao, 2015). EBV can establish life-long latent infections and, if it not cleared by the host immune system, it can contribute to cancer development, suggesting that it has evolved the ability to escape recognition by the host's innate immune system. Similarly, immunocompromised individuals, such as HIV and post-transplant patients, are prone to develop EBV-associated lymphomas, implying that when there is less host immune surveillance, the function of EBV changes. Since EBV pathogenesis consists of a virus-host interaction, both viral and host factors are involved in the viral immune evasion strategies. In primary EBV infections, cellular immunity is the main host immune response.

MicroRNAs (miRNAs) are a type of small non-coding RNA molecule, consisting of about 18 to 22 nucleotides. The mature single-stranded miRNAs bind to the 3'-untranslated region (UTR) of target mRNAs so as to inhibit the expression of target genes (Buchan and Parker, 2007; Jia et al., 2014). miRNAs have emerged as major transcriptional regulators of gene expression related to critical biological processes including cellular proliferation, differentiation, and viral immune evasion, and they are known to contribute to the development of cancers (Adams et al., 2014; Tsang and Tsao, 2015). In latent EBV infections involving host functional immunity, EBV expresses limited levels of protein but high levels of non-coding RNAs, including miRNAs. This indicates that the miRNAs are related to viral immune evasion (Jia et al., 2014; Zeng et al., 2014). EBV expresses about 40 mature miRNAs that target viral or cellular genes to induce different functions. In addition, viral proteins also regulate host miRNAs. EBV-encoded and EBV-regulated miRNAs appear to play an important role in immune evasion (Piedade and Azevedo-Pereira, 2016).

Accumulating studies have highlighted the link between the immune system and inflammatory tumor microenvironments. Tumors are surrounded and infiltrated by a variety of cell types, including fibroblasts, immune cells, and vascular endothelial cells, which can interact with each other to generate the tumor microenvironment. This complex microenvironment is thought to be regulated by the tumor cells in order to promote their survival and replication. The inflammatory tumor microenvironment plays a role in cancer development. EBV-encoded latent membrane proteins (LMP1 and LMP2A), which are able to induce the expression of multiple molecules linked to inflammation and the immune response, may act as targets of EBV-encoded miRNAs (Ressing et al., 2015). The molecules in the microenvironment constitute potential targets for anticancer and antiviral drug development (De Clercq and Li, 2016). This review will update the current knowledge about both EBV-encoded and EBV-dysregulated host-encoded miRNAs involved in viral immune evasion, the inflammatory response, and the tumor microenvironment in EBV-related carcinogenesis.

## BIOGENESIS OF EBV miRNAs

EBV was the first virus that encodes miRNAs to be discovered, and the biogenesis of these miRNAs is similar to the biogenesis of cellular miRNAs (Kim and Lee, 2012). The EBV miRNAs are encoded in two main clusters: the EBV *Bam*H I fragment H rightward open reading frame 1 (BHRF1) cluster and the *Bam*H I fragment A rightward transcript (BART) cluster. They are mainly transcribed by RNA polymerase II (pol II) to produce stem-loop structures, and they are subjected to sequential processing. The host endonuclease Drosha and DiGeorge syndrome chromosomal region 8 (DGCR8) complex cleaves the primary miRNA, resulting in a precursor miRNA, which has an imperfect hairpin structure and which is transported from the nucleus to the cytoplasm. Subsequently, the host protein Dicer further processes the precursor miRNA in the cytoplasm to generate the mature miRNA (Kim and Lee, 2012).

## EBV-ENCODED miRNAs REGULATE THE IMMUNE RESPONSE BY TARGETING VIRAL PROTEINS

EBV miRNAs are expressed in EBV-positive cells such as the primary effusion lymphoma cell line (PEL-1) and EBV-positive Burkitt's lymphoma and nasopharyngeal carcinomas (NPC). EBV BHRF1 encodes three miRNA precursors (EBV-miR-BHRF1-3), generating four mature EBV miRNAs. The BART region encodes 22 miRNA precursors (EBV-miR-BART1-22), producing about 40 mature miRNAs (Lo et al., 2012; Barth et al., 2011).

EBV miRNAs can target both cellular and viral mRNAs to evade the host immune response (as summarized in Figure 1, 2 and Table 1, 2). Viral proteins such as LMP1 and LMP2A have been found to be associated with EBV immune evasion (Ressing et al., 2015). LMP1 triggers multiple pathways such as the nuclear factor kappa-light-chain-enhancer of activated B cells (NF-kB), activator protein-1 (AP-1), Janus kinase (JAK)/signal transducer and activator of transcription (STAT) and phosphatidylinositol-specific phospholipase C (P1-PLC)-protein kinase C (PKC) signaling pathways to upregulate or downregulate a series of molecules related to the immune response (Dukers et al., 2000; Ding et al., 2007; Zheng et al., 2007). For example, LMP1 stimulates the receptor-interacting protein (RIP) and RIP-dependent K63-linked ubiquitination of interferon regulatory factor 7 (IRF7), which plays an important role in immune defense (Huye et al., 2007; Li et al., 2007). Four EBV miRNAs (miR-BART3, miR-BART5, miR-BART16, and miR-BART17) directly target LMP1, and thereby indirectly inhibit surface expression of certain immune co-receptors and adhesion molecules. This helps to alter the balance of LMP1's growth-promoting and pro-apoptotic actions (Lo et al., 2007; Skalsky et al., 2014; Verhoeven et al., 2016). Unlike the four abovementioned EBV miRNAs, miR-BART9 is involved in maintaining LMP1 mRNA stability in growing T cell lymphomas, though it does not target LMP1 directly (Lo et al., 2007). The targeting of LMP2A by miR-BART22 in EBV latent infections can lead to immune surveillance escape, cellular invasion, and metastasis (Lung et al., 2009; Kanda et al., 2015).

Figure 1. EBV miRNAs target viral and host genes related to the immune response. The viral miRNAs directly target immune molecules and indirectly target other host genes related to immunity to regulate the immune response. See Abbreviations for definitions of acronyms.

Figure 2. EBV-dysregulated host miRNAs. The red circles represent the upregulated miRNAs and the green circles represent the downregulated miRNAs. See Abbreviations for definitions of acronyms.

Table 1. EBV-encoded miRNAs that target viral proteins and cellular immune factors

| miRNAs | Targets | Functions | References |
|---|---|---|---|
| miR-BHRF1-2 | PRDM1 | B cell terminal differentiation | Ma et al., 2016 |
| | CTSB | Antigen processing | Albanese et al., 2016 |
| | IL-12B | T cell differentiation, activation, and recognition | |
| | MALT1 | Immune homeostasis | Wang et al., 2017 |
| miR-BHRF1-3 | CXCL-11 | Immunomodulation | Xia et al., 2008 |
| | TAP2 | Peptide transport | Albanese et al., 2016 |
| miR-BART1 | IL-12B | T cell differentiation, activation, and recognition | Albanese et al., 2016 |
| | IFI30 | Antigen processing | |
| miR-BART3 | LMP1 | Immune evasion and inflammation | Lo et al., 2007; Skalsky et al., 2014; Verhoeven et al., 2016 |
| miR-BART5 | | | |
| miR-BART16 | | | |
| miR-BART17-5p | | | |
| miR-BART2-5p | LGMN | Antigen processing | Albanese et al., 2016 |
| | CTSB | | |
| | IL-12B | T cell differentiation, activation, and recognition | |
| | MICB | Immune recognition | Diefenbach et al., 2000; Nachmani et al., 2009;Lisnic et al., 2010 |
| | BALF5 | Viral replication | Barth et al., 2008; Ma J. et al., 2016 |
| miR-BART3-3p | IPO7 | T cell activation and immune tolerance | Dolken et al., 2010 |
| miR-BART5 | PUMA | Apoptosis | Choy et al., 2008 |
| miR-BART8 | IFN-γ | Immunomodulation | Huang and Lin, 2014 |
| miR-BART10-3p | IL-12B | T cell differentiation, activation, and recognition | Albanese et al., 2016 |
| miR-BART11-5p | EBF1 | B cell differentiation | Ross et al., 2013 |
| miR-BART15 | NLRP3 | Inflammasome production | Haneklaus et al., 2012 |
| | BRUCE | Apoptosis | Choi et al., 2013 |
| miR-BART16 | CBP | Immunomodulation | Hooykaas et al., 2017 |
| miR-BART17 | TAP2 | Peptide transport | Albanese et al., 2016 |
| miR-BART20-5p | BAD | Apoptosis | Jung et al., 2014; Kim et al., 2015b |
| | IFN-γ | Immunomodulation | Huang and Lin, 2014 |
| | BRLF1 | Latent viral infection | Kim et al., 2015a |
| | BZLF1 | | |
| miR-BART22 | LMP2A | Immune evasion | Lung et al., 2009 |
| | NDRG1 | Immune surveillance escape | Kanda et al., 2015 |
| | IL12 | T cell differentiation, activation, and recognition | Albanese et al., 2016 |

Note: See Abbreviations for definitions of acronyms.

Table 2. Host miRNAs dysregulated by EBV latent proteins

| Viral proteins | Host miRNAs | References |
|---|---|---|
| EBNA1 | let-7a↑ | Onnis et al., 2012 |
| EBNA1 | miR-127↑ | Mansouri et al., 2014 |
| EBNA2 | miR-21↑ | Rosato et al., 2012 |
| EBNA3A/ EBNA3C | miR-221/ miR-222↑ | Bazot et al., 2015 |
| LMP1 | miR-203↓ | Yu et al., 2012 |
| LMP1 | miR-204↓ | Ma et al., 2014 |
| LMP1 | miR-15a↓ | Komabayashi et al., 2014 |
| LMP1 | miR-1↓ | Chen et al., 2015 |
| LMP1 | miR-183-96-182↓ | Oussaief et al., 2015 |
| LMP1 | miR-146a↑ | Zhao et al., 2012 |
| LMP1 | miR-10b↑ | Li et al., 2010a |
| LMP1 | miR-21↑ | Yang et al., 2013 |
| LMP1 | miR-155↑ | Yang et al., 2015 |
| LMP1 | miR-29b↑ | Anastasiadou et al., 2010 |
| LMP1 | miR-34a↑ | Forte et al., 2012 |
| LMP2A | miR-155↑ | Du et al., 2011 |

Note: ↑ indicates upregulation; ↓ indicates downregulation. See Abbreviations for definitions of acronyms.

In addition to LMP1 and LMP2A, other viral genes, such as early genes, may be targeted by EBV miRNAs in order to maintain the viral latent infection and inhibit lytic replication. EBV miR-BART2-5p targets BALF5, while miR-BART20-5p targets BZLF1 and BRLF1 (Barth et al., 2008; Jung et al., 2014). EBV miR-BART6-5p inhibits the expression of Epstein-Barr virus nuclear antigen 2 (EBNA2), which is a key activator that causes the latent infection to change to bring about lytic replication (Iizasa et al., 2010). Thus, it can be seen that EBV has evolved a set of miRNAs to effectively maintain the latent infection.

## EBV-ENCODED miRNAs REGULATE THE IMMUNE RESPONSE BY TARGETING CELLULAR IMMUNE-RELATED GENES

Viral miRNAs can also directly target cellular mRNAs involved in the host immune response (Figure 1). Both miR-BHRF1-3 and miR-BART17 target transporter associated with antigen processing 2 (TAP2), which is a mediator of antigenic peptide transportation and EBV epitope presentation (Albanese et al., 2016). EBV miR-BHRF1-2 has a role in the inhibition of PR domain-containing protein 1 (PRDM1) in lymphoblastoid cell lines (Ma et al., 2016). PRDM1 is a master regulator of B cell terminal differentiation and has been identified as a tumor suppressor gene in aggressive lymphomas such as diffuse large B cell lymphoma (DLBCL). The major histo compatibility complex class I-related chain B (MICB) is targeted by miR-BART2-5p, preventing natural killer (NK) cells from recognizing EBV-infected cells. In virus infections and tumor proliferation, MICB expression increases, and it has been found to activate NK and T cells, thereby initiating the immune reaction (Diefenbach et al., 2000; Nachmani et al., 2009; Lisnic et al., 2010; Liu et al., 2012). EBV miR-BART1, miR-BART2, and miR-BART22 co-target interleukin-12 (IL-12), leading to decreased T cell differentiation, activation, and recognition at different stages of infection and malignant disease (Albanese et al., 2016). EBV miR-BART3-3p weakens the cytotoxic effect by inhibiting the expression of importin 7 (IPO7), which regulates T cells activation and immune tolerance (Dolken et al., 2010). MiR-BART20-5p and miR-BART8 target and alter interferon-γ (IFN-γ) secretion in EBV-positive tumors (Huang and Lin, 2014). This may be why many immune cells (such as $CD8^+$ and $CD4^+$ T cells, B cells, NK cells, and macrophages) can be found in NPC, but EBV-specific cytotoxic T lymphocytes (CTLs) are incapable of increasing cell-mediated immunity.

In addition, CXC-chemokine ligand 11 (CXCL11) can be targeted by miR-BHRF1-3, which in turn shields EBV-infected B cells from CTLs (Xia et al., 2008). Recently, EBV miR-BHRF1-2-5p has been reported to target mucosa-associated lymphoid tissue lymphoma transport protein 1 (MALT1), a key regulator of immune homeostasis (Wang et al., 2017). EBV miR-BART16 interferes with the type I IFN signaling pathway by directly targeting cAMP response element-binding protein (CREB)-binding protein (CREBBP), a key transcriptional coactivator in IFN signaling (Hooykaas et al., 2017).

Therefore, EBV-encoded miRNAs impair the recognition and clearance functions of the host immune system, thus increasing viral pathogenesis. The identification of target genes of EBV miRNAs may help to unveil the role of EBV in immune evasion.

## LACK OF VIRAL miRNA GENE CLUSTERS IN THE EBV GENOME INCREASES THE HOST IMMUNE RESPONSE

EBV miRNAs are encoded by two gene clusters in the viral genome. The narrow spaces between the miRNAs indicate that there is interplay between them that influences their function and expression. The transcription order of the viral miRNAs remains unknown. A recent study found that the distance between two miRNAs in the same cluster may affect the miRNA expression levels, independent of the seed regions (Haar et al., 2016). The EBV BHRF1 miRNA cluster promotes B cell transformation. Increasing the distance between miR-BHRF1-2 and miR-BHRF1-3 in EBV enhances the expression of miR-BHRF1-3 but reduces the transforming potential of the BHRF1 miRNAs in B cells (Haar et al., 2016).

Therefore, studies on the miRNA clusters in the context of the whole viral genome may be valuable for elucidating the viral miRNA functions. To reach this goal, EBV mutants that lack the miRNA clusters may help. Several approaches have recently demonstrated that lack of the miRNA clusters in EBV mutants increases the $CD4^+$ and $CD8^+$ T cell host immune response, strongly supporting the conclusion regarding the important roles of EBV miRNAs in immune evasion.

Wolfgang Hammerschmidt's group has conducted multiple key studies on EBV mutants. Using EBV mutants that lacked the miRNA clusters, they demonstrated that the EBV miRNAs inhibit antiviral $CD4^+$ T cell responses by targeting IL-12 and peptide processing (Tagawa, 2016). At the same time, EBV miRNAs reduce immune surveillance by virus-specific $CD8^+$ T cells (Albanese et al., 2016). The findings are helpful to explain the abundance of miRNAs in the complex virus, and to clarify how EBV can escape elimination for the lifetime of its host in spite of intense adaptive host immune responses.

In another EBV mutant study, knock-out of the BHRF1 cluster increased the production of phosphatase and tensin homolog (PTEN) and p27, implying that BHRF1 miRNAs are associated with a propensity to promote neoplastic cell transformation, which is beneficial for long-term persistence of the virus in the host.

All these results support the importance of EBV miRNAs for immune evasion. EBV miRNAs use multiple distinct pathways, allowing the virus to evade host immune surveillance (Bernhardt et al., 2016). Understanding of the functions of EBV miRNAs provides novel insights into the elaborate systems that EBV has evolved to successfully establish lifelong infections. EBV miRNA-mediated interference may contribute to the maintenance of EBV-positive tumors.

## EBV LATENT PROTEINS DYSREGULATE HOST miRNAs TO REGULATE THE IMMUNE RESPONSE

In EBV-associated cancers, a few EBV latent proteins can be detected. These proteins (LMP1, LMP2A, EBNA1, EBNA2, EBNA3A, and EBNA3C) induce or inhibit the expression of a variety of endogenous miRNAs, which regulates the immune response (as summarized in Table 2 and Figure 2).

Multiple cellular miRNAs that are dysregulated by EBV can be induced in innate immune cells. Among them, miR-155, miR-146a, and miR-21 are associated with inflammation and immune modulation (Zhu et al., 2013; Zeng et al., 2015; Zhang et al., 2015a). MiR-155 suppresses Toll-like receptors (TLRs) and triggers an immune response (Sun et al., 2012). In the presence of a TLR3 ligand, the expression level of miR-21 can be upregulated in a NPC cell line (C666-1) and tissues (Miao et al., 2015). MiR155 also mediates an increase in IL-10 expression by B lymphocytes, resulting in the inhibition of $CD8^+$ T cell activation (Sun et al., 2012). By targeting C-C motif chemokine ligand 22 (CCL22), miR-34a is involved in immune escape and stromal cell recruitment (Yang et al., 2012). STAT3 can promote the expression of a large number of immunosuppressive factors such as IL-10 (Yu et al., 2016). In turn, these factors activate STAT3. STAT3 is able to transcriptionally bind to the promoter of miR-146a, which is a key regulator of innate antiviral responses (Sun et al., 2015). MiR-146a also targets two immune-associated genes, interleukin-1 receptor-associated kinase 1 (IRAK1) and TNF receptor-associated factor 6 (TRAF6). IRAK1 is a key innate immune signal molecule (Li et al., 2010b; Saba et al., 2014). It participates in signaling cascade regulation associated with TLR/IL-1R, changing the expression of inflammatory factors such as tumor necrosis factor-α (TNF-α) and type I IFNs. TRAF6 is involved in the cellular immune response by interacting with TLRs and the IFN-γ signal transduction pathway (Yang et al., 2007; Li et al., 2010b). Let-7a is upregulated by EBNA1 and has been shown to directly alter cell cycle progression and pro-inflammatory cytokine production. The let-7a expression and IL-6 production is significantly decreased by the depletion of E2F2 or NF-κB in immune-stimulated cells (Cho et al., 2015). MiR-222, miR-223, the miR-17~92 cluster, and miR-155 are associated with the differentiation of T cells (Riley et al., 2012; Poole and Sinclair, 2015; Saki et al., 2015). EBV-encoded EBNA2 induces the expression of miR-21, consequently promoting B cell transformation and driving the antiviral immune response (Rosato et al., 2012; Zhu et al., 2013). The upregulation of miR-127 in EBV-positive Burkitt's lymphoma may cause the downregulation of the B cell markers, B lymphocyte-induced maturation protein 1 (BLIMP-1) and X-box binding protein 1 (XBP-1) (Onnis et al., 2012).

These miRNAs dysregulated by EBV proteins may serve as the tumor promotors. EBV utilizes multiple host miRNAs to adapt to the host immune response and promote immune evasion. In-depth studies of this aspect may provide a more complete understanding about the pathogenic mechanism employed by EBV.

## EBV miRNAs SUPPRESS APOPTOSIS OF INFECTED CELLS TO MAINTAIN THE EBV LATENT STATUS AND IMMUNE EVASION

In general, EBV remains latent in tumors and expresses a limited repertoire of latent proteins to avoid host immune surveillance. Apoptosis may expedite EBV-associated tumor cell death and is detrimental for the viral latent infection and EBV evasion of the host immune attack. Thus, the virus has evolved strategies to suppress the apoptosis of infected cells. Several studies have directly or indirectly revealed that EBV miRNAs are involved in apoptosis suppression. For instance, in EBV-associated gastric carcinomas, miR-BART 20-5p not only targets the EBV immediate early genes BRLF1 and BZLF1, but also Bcl-2-associated death promoter (BAD) in the caspase-3-dependent apoptosis pathway (Jung et al., 2014; Kim et al., 2015).

In the BART cluster, an interference approach revealed that at least seven BART miRNAs are involved in anti-apoptotic activity in NPC by targeting multiple pro-apoptotic cellular genes. Naturally, the BHRF1 miRNA cluster is located at the BHRF1 gene, and the protein product of this gene has anti-apoptotic properties. If B cells are infected with a mutant EBV lacking the BHRF1 miRNAs, they may undergo apoptosis to a greater extent and grow more slowly compared to B cells infected with an intact virus. The BHRF miRNAs also regulate the expression of PTEN, p27, and a Bcl-2 homolog, allowing the expansion of the virus B cell reservoir during the early infection without increasing long-term immune pressure (Bernhardt et al., 2016). In NPC, miR-BART5 can target p53-upregulated modulator of apoptosis (PUMA) to inhibit cellular apoptosis (Choy et al., 2008). EBV BART miRNA Cluster I or II directly inhibit the pro-apoptotic protein, Bim (Marquitz et al., 2011). The translocase of outer mitochondrial membrane 22 (TOMM22), which serves as a receptor for the Bcl-2-associated X (BAX), is targeted by miR-BART16. Antisense knockdown of TOMM22 has been shown to inhibit the association of the pro-apoptotic protein Bcl-2-associated X (BAX) with mitochondria and thus prevent BAX-induced apoptosis (Bellot et al., 2007; Dolken et al., 2010). MiR-BART15-3p can induce apoptosis partially by inhibiting an apoptosis inhibitor, BIR repeat-containing ubiquitin-conjugating enzyme (BRUCE) (Choi et al., 2013). These reports show that EBV miRNAs help to maintain the viral latent status in the infected cells by preventing the host immune response.

## EBV REGULATES THE INFLAMMATORY TUMOR MICROENVIRONMENT VIA miRNAs

The regulation of cellular or viral protein expression by EBV-encoded and EBV-regulated miRNAs may result in changes in cellular pathways, creating a virus-friendly microenvironment. LMP1 regulates the tumor microenvironment by inducing the NF-κB signaling pathway (Yu et al., 2012). As mentioned above, four EBV BART-miRNAs co-target LMP1, potentially contributing to the modulation of the tumor microenvironment. It has also been shown that EBV-miR-BART11 targets forkhead box P1 (FOXP1), a key molecule involved in differentiation of monocytes to macrophages (Song et al., 2016). In EBV-associated NPC and gastric carcinomas, the inhibition of FOXP1 induces the differentiation of tumor-associated macrophages (TAMs) and secretion of inflammatory cytokines into the tumor microenvironment.

EBV miR-BART15 targets the NOD-like receptors protein 3 (NLRP3) and inhibits the inflammasome production of IL-1β (Haneklaus et al., 2012). NLRP3 is one of the best-studied NLRs, which monitor intracellular homeostatic molecules. Many cell types require NLRP3 inflammasome priming, such as stimulation with a TLR ligand. As mentioned above, EBV-related miRNAs influence IL-6 signaling. IL-6, a pro-inflammatory factor secreted by immune cells, is able to promote the secretion of miR-21 and miR-29b by tumor cells, and the miRNAs in turn induce the expression of IL-6 (Patel and Gooderham, 2015). Downregulation of IPO7 by miR-BART3 in macrophages has been shown to reduce IL-6 production upon lipopolysaccharide challenge (Yang et al., 2009). In the pathogenesis of inflammatory diseases (including tumors and infections), miR-21 can act as an immune response circuit switch in order to control the balance between the initial pro-inflammatory response and the anti-inflammatory response (Sheedy, 2015).

Matrix metalloproteinase-2 and -9 (MMP2 and MMP9), which are miR-29b targets, may be involved in extracellular matrix remodeling and inhibition of epithelial-mesenchymal transition (Chou et al., 2013). The miR-203 inhibited by EBV-LMP1 targets IL-8, which is a chemotaxis cytokine and immune response mediator (Yu et al., 2011; Yu et al., 2012; Qu et al., 2015). EBV-induced miR-155 and miR-146a are also important molecules in the regulation of the inflammatory reaction (Zhang et al., 2015a; Yang et al., 2016; Bitar et al., 2017). EBNA1-induced miR-let-7a is a potential mediator of the inflammatory tumor microenvironment because miR-let-7a reduces the expression of inducible nitric oxide synthase (iNOS) and IL-6, and enhances the expression of IL-4 and IL-10 in microglia (Chen et al., 2007; Mansouri et al., 2014; Cho et al., 2015).

Cells can secrete multiple types of membrane vesicles, including exosomes, which are the best-characterized membrane vesicles. Exosomes are vesicles of 50–100 nm in size that are over-produced by most proliferating cell types during normal states, pathological states (such as cancer), and states such as pregnancy. Exosomes contain a wide variety of proteins, lipids, RNAs, non-transcribed RNAs, miRNAs, and small RNAs (Yin et al., 2013; Becker et al., 2016). Ongoing efforts have shown that miRNAs are secreted into tumor microenvironments to regulate cancer cell proliferation, migration, immune response, intercellular communication, and stromal modification, thereby promoting tumor growth and progression (Meckes et al., 2010; Zhang et al., 2015b; Li et al., 2016). EBV-regulated miRNAs and viral proteins can be secreted in exosomes to regulate the tumor microenvironment. It is speculated that tumor cells inhibit the host immune response by transporting exosomes containing EBV miRNAs to nearby cells or immune cells.

EBV-positive lymphoblastoid cell lines and NPC cells contain EBV BART miRNAs. The BART miRNAs secreted by NPC cells have enough stability to diffuse from the tumor site to the peripheral bloodstream, as well as to other non-infected cells. In EBV-positive NPC, exosomes containing several viral miRNAs, including miR-BART4, miR-BART7, miR-BART16, miR-BART9, miR-BART12 and miR-BART13, have been shown to be transported to adjacent endothelial cells (Pegtel et al., 2010). EBV-transformed lymphoblastoid B cells also secrete several viral miRNAs, including miR-BHRF1-1, miR-BHRF1-2, miR-BART1-3p, miR-BART1-5p, and miR-BART2-5p (Meckes et al., 2010). EBV-infected B lymphocytes are able to secrete miR-BART15 (Choi et al., 2013). The exosomal miR-BART15 provides a favorable microenvironment for the growth of EBV-associated tumors. This evidence shows that miRNAs are utilized by EBV for cell-to-cell communication and expanding the influence of the virus.

As described above, some viral proteins such as LMP1 are regulators of the immune response. LMP1 can be packaged into exosomes (Ceccarelli et al., 2007). Exosomes can deliver viral proteins to recipient cells such as epithelial, endothelial, and fibroblast cells (Meckes et al., 2010). It is noteworthy that LMP1 is not expressed in all the cells of EBV-associated tumors (Haneklaus et al., 2012). However, through exosomal transmission, viral oncoproteins can induce immune dysfunction in neighboring or even distant EBV-negative cells, providing a favorable microenvironment for the growth and migration of EBV-associated cancers.

## EBV-ENCODED AND EBV-DYSREGULATED miRNAs ARE POTENTIAL BIOMARKERS AND THERAPEUTIC TARGETS

In the above mentioned miRNA studies, a series of EBV-encoded and EBV-dysregulated miRNAs have been shown to be potential clinical targets. EBV miR-BART1-5p and BART1-3p are dramatically increased in the N2-3 stages or advanced stages (III–IV) of NPC (Cai et al., 2015; Zheng et al., 2016). The levels of both miR-BART7 and miR-BART13 in NPC plasma are found to be present at distinct levels among patients with different stages of NPC, with elevated levels among patients with advanced disease. An analysis of 41 NPC patients before and after radiotherapy showed that miR-BART7 and miR-BART13 were diminished after treatment, suggesting a potential use for them in terms of NPC serological diagnosis and prediction of treatment efficacy (Zhang et al., 2015). The overexpression of EBV-miRBART10-3p in patients with NPC is significantly associated with poor disease-free and overall survival (Yan et al., 2012). These findings suggest that the effectiveness of current NPC treatments may be a result of the modulation of miRNA expression. In addition, miR-BART1-5p, miR-BART4-5p, and miR-BART20-5p are readily detectable in EBV-associated gastric carcinoma tissues, and the expression level of BART20-5p may predict relapse among these patients (Kang et al., 2017).

A study revealed a significant correlation between miR-183 and the NPC N stages (Tang et al., 2012). In 16 patients with non-keratinizing undifferentiated NPC, the miR-146a expression level was significantly elevated and may be useful as an indicator of NPC development (Zhao et al., 2012). Low expression of miR-204 is closely correlated with EBV infection status, lymph node metastasis, and more advanced clinical stages (Ma et al., 2014). There is a negative correlation between plasma miRNA expression and cancer progression, with miR-21 expression levels being significantly associated with the NPC T and N stages (Liu et al., 2013). In serum samples from patients with DLBCL, miR-21 expression levels are positively correlated with poor overall survival, thereby acting as an independent prognostic marker of overall survival in patients with DLBCL (Li et al., 2015). As described above, miR-155 has a crucial role in the immune response and inflammation. The miR-155 is upregulated in several malignancies and is associated with poor prognosis, thus it may be useful as a diagnostic and prognostic biomarker (Hanne et al., 2016; Due et al., 2016).

In addition, functional miRNAs in exosomes have been recognized as potentially stable cancer biomarkers. The miR-21 is found in many types of exosomes including tumor- and immunocyte-derived exosomes (Fabbri et al., 2012; Tian et al., 2014). Exosomal miRNAs such as miR-BART7-3p are usually stable, making them potentially ideal biomarkers (Gourzones et al., 2010). EBV BART miRNAs are secreted by NPC cells, with enough stability to diffuse from the tumor site to the peripheral bloodstream at levels that are detectable in plasma samples. Our increasing knowledge about the role of miRNAs in immune evasion will prove useful for understanding EBV persistence and developing improved treatments for EBV-associated cancers and other diseases.

## CONCLUSIONS

EBV miRNAs are able to change the expression of a series of genes to regulate immune evasion. They also play a powerful role in regulating cancer progression via construction and modification of the microenvironment. EBV-encoded and EBV-dysregulated miRNAs are critical regulators of cellular gene expression associated with innate and adaptive immunity. The information presented in this review is summarized in Figure 3. Emerging evidence has demonstrated that exosomes play important roles in intracellular communication associated with cancer development. EBV-positive tumor cells secrete exosomes containing EBV-associated molecules such as miRNAs and viral proteins. Further studies of EBV-encoded and EBV-regulated miRNAs will be of great significance for the identification of the in-depth immunological mechanisms underlying EBV pathogenesis. The development of new treatments should place more emphasis on identifying potential target miRNAs to improve the effectiveness and personalization of EBV treatment.

## ABBREVIATIONS

BAD, Bcl-2-associated death promoter; BALF5, Viral DNA polymerase gene; BRLF1, EBV immediate early gene; BRUCE, BIR repeat-containing ubiquitin-conjugating enzyme; BZLF1, EBV immediate early gene; CBP, CREB binding protein; CCL17, Chemokine (C-C motif) ligand 17; CCL22, Chemokine (C-C motif) ligand 22; CTSB, Cathepsin B; CXCL-11, CXC-chemokine ligand 11; DGCR8: DiGeorge syndrome chromosomal region 8; DNAM-1, CD226 molecule; EBF1, Early B-cell factor 1; EBNAs, Epstein-Barr virus nuclear antigens; EBNA1, Epstein-Barr virus nuclear antigen 1; EBNA2, Epstein-Barr virus nuclear antigen 2; EBNA3A/EBNA3C, Epstein-Barr virus nuclear antigen 3A/3C; ICAM, Intercellular adhesion molecule; IFI30, Lysosomal thiol reductase; IFN-β: Interferon-β; IFN-γ: Interferon-γ; IFN-α: Interferon-α; IL-1β, Interleukin-1β; IL-6, Interleukin-6; IL-8, Interleukin-8; IL-10, Interleukin-10; IL-12, Interleukin-12; IL-12β, Interleukin-12 subunit beta; IPO7, Importin-7; IRF3, Interferon regulatory factor 3;

Figure 3. Schematic for the immune evasion mechanism mediated by EBV-encoded and EBV-dysregulated host-encoded miRNAs. In EBV-infected epithelial tumors, EBV miRNA clusters utilize multiple host proteins (such as Drosha, DGCR8, and Dicer) to process the viral miRNAs. To promote immune evasion, the EBV miRNAs target host genes to regulate immune-related gene function, the $CD4^+$ and $CD8^+$ T cellular response, apoptosis, and the inflammation and reconstitution of the tumor microenvironment. In addition, EBV miRNAs directly target viral genes to regulate their expression. The proteins encoded by EBV genes can regulate the expression of multiple host miRNAs to activate or inhibit their functions and thereby promote immune evasion. EBV-positive tumor cells secrete exosomes containing EBV-encoded and EBV-dysregulated host miRNAs to contribute to the inflammation and reconstitution of the tumor microenvironment. See Abbreviations for definitions of acronyms.

IRF7, Interferon regulatory factor 7; LGMN, Legumain; LMP1, Latent membrane protein 1; LMP2A, Latent membrane protein 2A; MALT1, Mucosa-associated lymphoid tissue lymphoma translocation protein 1; MICA, MHC class I polypeptide-related sequence A; MICB, Major histocompatibility complex class I-related chain B; NDRG1, N-myc downstream-regulated gene 1; NLRP3, Nod-like receptor protein 3; TAP2, Transporter 2; PDL-1, Programmed cell death 1 ligand 1; PRDM1, PR domain zinc finger protein 1; PUMA, A p53 upregulated modulator of apoptosis.

## ACKNOWLEDGMENTS

This work was supported by the National Natural Science Foundations of China (81372139, 31670171), the Hunan Provincial Natural Science Foundation of China (2015 JJ2149), and the Hunan Provincial Innovation Foundation for Postgraduates (CX2016B055).

## COMPLIANCE WITH ETHICS GUIDELINES

The authors declare that they have no conflict of interest. This article does not contain any studies with human or animal subjects performed by any of the authors.

## OPEN ACCESS

This article is distributed under the terms of the Creative Commons Attribution 4.0 International License (https://creativecommons.org/licenses/by/4.0/), which permits unrestricted use, distribution, and reproduction in any medium, provided you give appropriate credit to the original author(s) and the source, provide a link to the Creative Commons license, and indicate if changes were made.
